# KPC-Producing *Enterobacterales* from Douro River, Portugal—Persistent Environmental Contamination by Putative Healthcare Settings

**DOI:** 10.3390/antibiotics12010062

**Published:** 2022-12-29

**Authors:** Josman Dantas Palmeira, Inah do Arte, Mai Muhammed Ragab Mersal, Catarina Carneiro da Mota, Helena Maria Neto Ferreira

**Affiliations:** 1 UCIBIO—Applied Molecular Biosciences Unit, REQUIMTE, University of Porto, 4050-313 Porto, Portugal; 2 Laboratory of Microbiology, Biological Sciences Department, Faculty of Pharmacy, University of Porto, 4050-313 Porto, Portugal; 3 Associate Laboratory i4HB-Institute for Health and Bioeconomy, University of Porto, 4050-313 Porto, Portugal; 4 CESAM—Centre for Environmental and Marine Studies, University of Aveiro, 3810-193 Aveiro, Portugal; 5 PICTIS—International Platform for Science, Technology and Innovation in Health, University of Aveiro (Portugal) & FIOCRUZ, Rio de Janeiro 1040-360, Brazil

**Keywords:** carbapenemases, KPC, *Enterobacterales*, environmental contamination, river water

## Abstract

Carbapenemase-producing *Enterobacterales* (CPE) are a growing concern, representing a major public health threat to humans, especially in healthcare settings. In the present study, we evaluated the persistent contamination by carbapenem-resistant *Enterobacterales* in water from Douro River, Portugal. KPC-producing *Enterobacterales* were detected in five water samples separated chronologically by 15 days each. Susceptibility testing was performed by disk-diffusion-method according to Clinical and Laboratory Standards Institute (CLSI), phenotypic carbapenemase activity was evaluated by carbapenem inactivation method, presumptive identification of the isolates was performed by CHROMagar orientation and confirmed by API-20E. Carbapenemase genes were screened by PCR and the clonality of all isolates was assessed by *XbaI*-Pulsed Field Gel Electrophoresis (PFGE). Fifteen KPC-producing *Enterobacterales* isolates were selected, identified as multidrug-resistant and showed a resistance profile to non-beta-lactam antibiotics: sulfamethoxazole + trimethoprim (7/15), ciprofloxacin (3/15), fosfomycin (3/15) and chloramphenicol (2/15). Isolates were identified as (6) *Escherichia coli* and (9) *Klebsiella pneumoniae*. Our results suggest a punctual contamination with KPC-producing *Enterobacterales* continued through the time. The absence of clonality between the isolates suggests a circulation of mobile genetic element harbouring KPC gene in the origin of contamination. This work provides a better understanding on the impacts of water pollution resulting from human activities on aquatic environments.

## 1. Introduction

The emergence of carbapenemase-producing *Enterobacterales* (CPE) has been identified in recent years as one of the major public-health concerns over the last decade. Carbapenems constitute the last-resort class of antibiotics for nosocomial infections, as in some cases of infection there are very limited treatment options available, being associated with mortality rates that usually exceed 40% [1,2,3,4]. CPE are no longer restricted to healthcare settings, being detected in different ecological niches, such as river waters [5].

In Portugal, KPC prevails over other carbapenemases, with KPC-2 being the most prevalent in northern Portugal and KPC-3 being more associated with healthcare settings [6,7,8].

In 2015, as result of multidrug-resistant pathogens, particularly *Klebsiella pneumoniae* carbapenemase-producing *Enterobacterales* (KPC-E), approximately 33,000 people died in Europe. Nowadays, KPC-E is a concern due to the high level of endemicity observed in various areas worldwide, being considered as an urgent threat and one of the biggest global public-health challenges [9].

It is known that there are several connections between the environmental, human and animal compartments that allows for the movement of bacteria, as well as mobile genetic elements and antibiotic resistance [10]. The aquatic environment is often exposed to discharges from various sources, through inadequate handling in agriculture, healthcare settings and industry, collecting biological and chemical contaminants. This allows this ecosystem to perform as a vehicle for antibiotic-resistant bacteria and antibiotic resistance genes that circulate and spread in the environment, reaching and affecting humans [11,12]. It is worth noting that river ecosystems are very relevant, playing key roles in ecosystem functions such as drinking water, food, agriculture, animal production and leisure activities [13].

Presently, there are few epidemiologic studies regarding CPE recovered from rivers. The aim of this study was to explore the presence of CPE in the ecosystem of Douro river, an important river in the Porto metropolitan area and hence with a considerable anthropogenic impact and scarce contribution of animal faecal contaminants.

## 2. Results

### 2.1. Carbapenemase-Producing Enterobacterales

#### 2.1.1. Identification and Antimicrobial Resistance Phenotypes

KPC-producing *Enterobacterales* were selected in all five water samples analysed (Table 1). Isolates were identified as six *Escherichia coli* and nine *Klebsiella pneumoniae*. All identified bacteria presented were resistant to meropenem and were multi-drug resistant (MDR) according to the definition by Magiorakos et al. [14]. A substantial proportion of isolates showed reduced susceptibility to non-beta-lactam antibiotics: sulfamethoxazole + trimethoprim (7/15, 46.7%), ciprofloxacin (3/15, 20%), fosfomycin (3/15, 20%) and chloramphenicol (2/15, 13.3%). No resistance was observed for tobramycin and netilmicin, with all isolates displaying a profile of susceptibility or intermediate.

#### 2.1.2. Carbapenemases and ESBL

Fifteen isolates producing carbapenemases were confirmed by the carbapenem inactivation method (CIM) and the carbapenemase phenotype was due to the presence of *bla*_KPC_ genes (15/15). No other carbapenemase genes were detected.

More, all of the isolates additionally carried 1 to 3 of the most prevalent genes of ESBL-producing bacteria. Seven isolates carried *bla*_CTX-M group 2_ (7/15, 46.7%), *bla*_TEM_ was detected in six isolates (6/15, 40%) and *bla*_OXA_ in four isolates (4/15, 26.7%). Both *bla*_CTX-M group 9_ and *bla*_SHV_ were not detected in any of the isolates.

#### 2.1.3. Clonal Relation of KPC-Producing *Enterobacterales*

No clonal relation was detected in the *E. coli* or in the *K. pneumoniae* evaluated (Figure S1). With exception of 3 *E. coli* isolates with similar pattern, which were isolated in the times D0, D30 and D60.

## 3. Discussion

Nowadays, anthropogenic activities may be one of the biggest reasons behind the dissemination of carbapenem resistance [15]. In this study, we describe the presence of KPC-producing *Enterobacterales*, in water, over time in Douro River, Vila Nova de Gaia, for 2 months.

CPE are more confined to healthcare settings, including occurrences of outbreaks, however, reports of these *Enterobacterales* have been increasingly reported in the environment [16]. All of the 15 isolates detected were classified as MDR, showing resistance to three or more classes of antibiotics in addition to meropenem resistance, which highlights an alarming resistance of these *Enterobacterales* strains. These MDR *Enterobacterales* are a public health concern due to their therapeutic importance, as the antibiotics to which they are resistant are some of the main therapeutic choices in the hospitals, particularly carbapenem resistant, which is mostly by transference of *bla*_KPC_ [17,18]. These isolates were identified as 6 *E. coli* and 9 *K. pneumoniae*, all of them carrying *bla*_KPC_. As expected, *bla*_KPC_ prevalence was higher in *K. pneumoniae* than *E. coli*. Data from the literature reports that *Klebsiella* spp. and *E. coli* are the most common carbapanemase producing *Enterobacterales* isolated from rivers and *bla*_KPC_ the most prevalent in Europe [3,19], which is in line with our results.

*K*. *pneumoniae* is one of the pathogens that were given the highest “priority status”, due to its potential drug resistance, including carbapenems [19]. In Portugal, an increasing trend of carbapenem resistance in *K. pneumoniae* has been described, evolving from 5.2% in 2016 to 11.6% in 2020, as reported by the European Centre for Disease Prevention and Control (ECDC) [20]. KPC-producing *K. pneumoniae* were the most prevalent in our study (9/15). Isolates harbouring these gene seem to be widespread in Portuguese hospitals [6,7]. Thus, the presence of *bla*_KPC_-harbouring *K. pneumoniae* in this study is in line with the epidemiology reported in Portuguese healthcare settings, which suggests an anthropogenic origin as the source of contamination.

*E. coli* are predominantly harmless bacteria that colonize the normal gut flora of humans, yet they are genetically diverse bacteria than can lead to extraintestinal infections [21,22]. Thus, the presence of *E. coli* resistant to carbapenems may severely affect human health, since it is known its capacity to carry several virulence factors and genes that are not present in strains belonging to commensal strains phylogroups [22]. The occurrence of *E. coli* KPC-producing strains in the aquatic environment is a worry, being distributed worldwide, although it is scarce, and it is described in countries with high rates of this carbapenemase in *K. pneumoniae* [18]. The emergence of *bla*_KPC_ in *E. coli* has been linked to many mechanisms, such as plasmid exchange with other *Enterobacterales*, the global spread of genetically related strains and transpositional events in the species [18]. Therefore, the spread of *E. coli* carbapenem-resistant has severe implications in infection control, once compared with other CPE, since *E. coli* is a common bacteria in the community and healthcare settings and is easily transmitted.

KPC-producing *Enterobacterales* have been recurrently reported in hospitals sewage [5,16] and areas where industry is developed, such as agroindustry [3]. However, in our study, in a location as our sampling site, which is an important touristic spot where none of the above activities are practiced near, it is unusual and worrisome to find these reports. Fifteen KPC-producing *Enterobacterales* isolates were detected in this work with no clonal relationship, away from healthcare settings. The results suggest that the persistence of KPC-producing *Enterobacterales* was not related to clonal persistence. PFGE analysis showed the absence of a clonal relationship in the 15 isolates, which leads us to believe in the existence of a mobile genetic element responsible for the transmission of *bla*_KPC_ between the different isolates. KPC-producing bacteria are one of the biggest challenges in healthcare settings worldwide [23]. The description of this public health threat in urban river waters, suggests environmental contamination by accidental healthcare setting sewage, once KPC-producing bacteria are characteristic of healthcare associated intestinal colonization. Our results suggest a punctual contamination with KPC-producing *Enterobacterales* continued through the time. The absence of clonality between the isolates suggests a circulation of mobile genetic element harbouring KPC gene in the origin of contamination. Gut colonization of the community population does not show this kind of threats and contamination of other zones of the river show contamination with coliforms but without expression of carbapenemases [24,25,26,27].

## 4. Materials and Methods

### 4.1. Sampling and Bacterial Isolation

Chronologically separated by 15 days (D0, D15, D30, D45, D60), five samples of water of Douro River were collected in the same place in Vila Nova de Gaia in 2021, from January to March.

The samples were stored directly in sterile glass bottles, held in the refrigerator and analysed shortly after collection (1 to 3 h).

We filtered 100 mL of the water samples, in duplicate, through sterile cellulose acetate membranes with 0.2 μm pore size (Frisenette, Knebel, Denmark) and placed them in MacConkey Agar (Liofilchem, Roseto degli Abruzzi, Italy) supplemented with meropenem (MRP, 0.5 µg/mL, Sigma-Aldrich, Darmstadt, Germany) to select carbapenem-resistant isolates [28]. Thereafter, the plates were incubated overnight at 37 °C. MacConkey Agar (Liofilchem, Roseto degli Abruzzi, Italy) without antibiotic, was used as growth control and control of the culture media was accomplished using *Escherichia coli* ATCCC 25922, a control strain.

### 4.2. Bacterial Identification and Antimicrobial Susceptibility Testing

One colony per morphology was picked from selective media onto the antibiotic-selection plate and incubated at 37 °C for 24 h.

The antimicrobial resistance profiles of the isolated bacteria were evaluated with 17 antibiotics by the agar diffusion method, according to Clinical and Laboratory Standards Institute (CLSI) [29]. Using antibiotic disks (Oxoid, Basingstoke, UK and Liofilchem, Roseto degli Abruzzi, Italy) for the following antibiotics: amoxicillin (AML, 10 μg), amoxicillin and clavulanic acid (AMC, 30 μg), aztreonam (ATM, 30 μg), cefepime (FEP, 30 μg), cefotaxime (CTX, 30 μg), cefoxitin (FOX, 30 μg), ceftazidime (CAZ, 30 μg), meropenem (MRP, 10 μg), nitrofurantoin (FUR, 300 μg), ciprofloxacin (CIP, 5 μg), fosfomycin (FOT, 200 μg), tobramycin (TOB, 10 μg) tetracycline (TET, 30 μg), levofloxacin (LEV, 5 μg), chloramphenicol (CHL, 30 μg), sulfamethoxazole + trimethoprim (SXT, 25 μg) and netilmicin (NET, 10 μg).

Phenotypic carbapenemase activity was evaluated by carbapenem inactivation method (CIM) which was performed on isolates showing reduced susceptibility to carbapenems. For a quick presumptive identification, CHROMagar Orientation (CHOROMagar, Paris, France) was performed and this identification was further confirmed with API 20E (Biomérieux, Marcy l’Etoile, France).

### 4.3. Beta-Lactamase Genes Screening

The bacterial DNA of carbapenem-resistant isolates was screened for carbapenemase genes. PCRs multiplexes were performed, which include the most prevailing acquired carbapenemase genes (*bla*_KPC_, *bla*_OXA-48_, *bla*_NDM_, *bla*_VIM_ and *bla*_IMP_). The PCR was executed under the followed conditions: 10 min at 94 °C, followed of 36 cycles of 30 s at 94 °C, 40 s at 52 °C and 50 s at 72 °C and a final extension of 5 min at 72 °C [30], using Super Hot Master Mix (Bioron, Römerberg, Germany).

Additionally, PCR multiplex was also performed for the most prevailing ESBL genes, *bla*_CTX-M group 1_, *bla*_CTX-M group 2_, *bla*_CTX-M group 8_, *bla*_CTX-M group 9_, *bla*_CTX-M group 25_, *bla*_TEM_, *bla*_OXA_ and *bla*_SHV_. For *bla*_CTX-M_, the amplification conditions were initial denaturation at 94 °C for 5 min, 30 cycles of 94 °C for 25 s, 52 °C for 40 s and 72 °C for 50 s and 6 min at 72 °C [31]. Regarding *bla*_TEM_, *bla*_OXA_ and *bla*_SHV_, the amplification was carried out at 94 °C for 10 min, 30 cycles of 94 °C for 40 s, 60 °C for 40 s, 72 °C for 1 min and a final elongation at 72 °C for 7 min [32].

### 4.4. Pulsed-Field Gel Electrophoresis

For clonality evaluation, PFGE was performed according to PulseNet USA standard protocol, through a CHIF-DR III system (BIO-RAD-U.S.A., Hercules, CA, USA) using 10 s as initial time and 40 s as final time for 21 h of running, after digestion of immobilized DNA with *XbaI* restriction enzyme (Bioron, Römerberg, Germany) [33].

## 5. Conclusions

In conclusion, our findings highlight the importance of AMR research in environmental samples and further analysis should be made to detect the possible causes of the presence and persistence of CPE in single specific location of this urban river. These results suggest a focal contamination, since it prevailed in the five samples, collected chronologically and separated by 15 days.

This work shows the relevance of this finding as a powerful biological marker of focal contamination and their persistence in river environments, by putative healthcare setting wastewaters, which may be related to the location and circulation of these genes by mobile genetic elements. These results alert to the need to expand the monitorization of carbapenemases dispersion in bacteria, particularly in natural environments exposed to anthropogenic pressures.

## Figures and Tables

**Table 1:** Identification of the 15 isolates per sample, bacterial specie, carbapenemase gene, other beta-lactamases and non-beta-lactamases resistance.

Sample	Isolate ID	Bacterial Species	Carbapenemase Gene	Other Beta-Lactamases	Non-Beta-Lactam Resistance
D0	1-MEM-A	*E. coli*	*bla*_KPC_	*bla*_CTX-M-group 2_	CIP, SXT
D0	1-MEM-B	*E. coli*	*bla*_KPC_	*bla*_CTX-M-group 2_	CIP
D0	1-MEM-D	*K. pneumoniae*	*bla*_KPC_	*bla*_CTX-M-group 1_	-
D0	1-MEM-D(2)	*E. coli*	*bla*_KPC_	*bla*_CTX-M-group 2_	FOT
D15	1(2)-MEM-A	*K. pneumoniae*	*bla*_KPC_	*bla*_TEM_, *bla*_OXA_	SXT
D15	1(2)-MEM-C	*K. pneumoniae*	*bla*_KPC_	*bla*_CTX-M-group 8_	SXT
D30	1(3)-MEM-B	*E. coli*	*bla*_KPC_	*bla*_CTX-M-group 2_	SXT, FUR
D30	1(3)-MEM-D	*K. pneumoniae*	*bla*_KPC_	*bla*_CTX-M-group 2_, *bla*_TEM_, *bla*_OXA_	-
D45	1(4)-MEM-A	*K. pneumoniae*	*bla*_KPC_	*bla*_TEM_	FOT
D60	1(5)-MEM-A	*K. pneumoniae*	*bla*_KPC_	*bla*_CTX-M-group 1_, *bla*_TEM_, *bla*_OXA_	CIP, LEV, TET, SXT, CHL, FUR
D60	1(5)-MEM-B	*K. pneumoniae*	*bla*_KPC_	*bla*_OXA_	-
D60	1(5)-MEM-C	*K. pneumoniae*	*bla*_KPC_	*bla*_TEM_	LEV, CHL
D60	1(5)-MEM-D	*K. pneumoniae*	*bla*_KPC_	*bla*_CTX-M-group 2_, *bla*_TEM_	SXT
D60	1(5)-MEM-E	*E. coli*	*bla*_KPC_	*bla*_CTX-M-group 2_	-
D60	1(5)-MEM-F	*E. coli*	*bla*_KPC_	*bla*_CTX-M-group 1_	SXT, FOT

Ciprofloxacin (CIP), levofloxacin (LEV), tetracycline (TET), sulfamethoxazole + trimethoprim (SXT), chloramphenicol (CHL), fosfomycin (FOT) and nitrofurantoin (FUR).

## Data Availability

Not applicable.

## Supplementary Materials

The following supporting information can be downloaded at: https://www.mdpi.com/article/10.3390/antibiotics12010062/s1, Figure S1: PFGE fingerprint of KPC-producing *E. coli* (a) and *K. pneumoniae* (b) from river Douro waters.
