# Supplementary material for: KPC-Producing Enterobacterales from Douro River, Portugal—Persistent Environmental Contamination by Putative Healthcare Settings
Source: Antibiotics (Basel). 2022 Dec 29;12(1):62. doi: 10.3390/antibiotics12010062 (PMC9855090; doi:10.3390/antibiotics12010062)
Supplement: Supplementary file 1 [file antibiotics-12-00062-s001.zip › antibiotics-2114234-supplementary.pdf]

SUPLEMENTAR MATERIAL

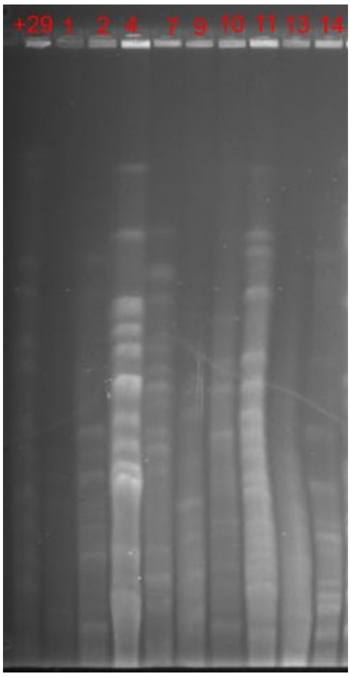

4 = 1(2)-MEM-A
7 = 1-MEM-D
9 = 1(3)-MEM-D
10 = 1(4)-MEM-A
11 = 1(5)-MEM-D
13 = 1(5)-MEM-B
14 = 1(5)-MEM-C

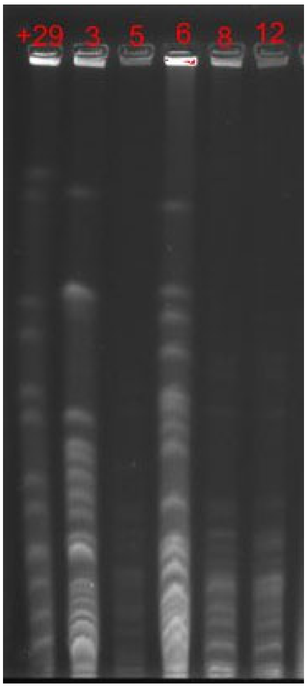

3 = 1-MEM-A
5 = 1(3)-MEM-B
6 = 1(5)-MEM-F
8 = 1-MEM-B
12 = 1(5)-MEM-E

Was not possible to determine the PFGE pattern for the isolates 1(2)-MEM-C and 1(5)-MEM-A of *K. pneumoniae* and 1-MEM-D(2) of *E. coli*

Figure S1. PFGE pattern of KPC-producing *K. pneumoniae* 9 (a) and *E. coli* 6 (b) from river Douro waters.
